# Design and Simulation of Material-Integrated Distributed Sensor Processing with a Code-Based Agent Platform and Mobile Multi-Agent Systems

**DOI:** 10.3390/s150204513

**Published:** 2015-02-16

**Authors:** Stefan Bosse

**Affiliations:** University of Bremen, Dept. of Mathematics & Computer Science, Robert Hooke Str. 5, 28359 Bremen, Germany; E-Mail: sbosse@uni-bremen.de; Tel.: +49-421-17845-4103

**Keywords:** sensor networks, multi-agent system, code morphing, stack machines, distributed computing, agent-based platform and network simulation

## Abstract

Multi-agent systems (MAS) can be used for decentralized and self-organizing data processing in a distributed system, like a resource-constrained sensor network, enabling distributed information extraction, for example, based on pattern recognition and self-organization, by decomposing complex tasks in simpler cooperative agents. Reliable MAS-based data processing approaches can aid the material-integration of structural-monitoring applications, with agent processing platforms scaled to the microchip level. The agent behavior, based on a dynamic activity-transition graph (ATG) model, is implemented with program code storing the control and the data state of an agent, which is novel. The program code can be modified by the agent itself using code morphing techniques and is capable of migrating in the network between nodes. The program code is a self-contained unit (a container) and embeds the agent data, the initialization instructions and the ATG behavior implementation. The microchip agent processing platform used for the execution of the agent code is a standalone multi-core stack machine with a zero-operand instruction format, leading to a small-sized agent program code, low system complexity and high system performance. The agent processing is token-queue-based, similar to Petri-nets. The agent platform can be implemented in software, too, offering compatibility at the operational and code level, supporting agent processing in strong heterogeneous networks. In this work, the agent platform embedded in a large-scale distributed sensor network is simulated at the architectural level by using agent-based simulation techniques.

## 1. Introduction and State-of-the-Art

Structural monitoring of mechanical structures allows deriving not just loads, but also their effects on the structure, its safety and its functioning from sensor data. A load monitoring system (LM) can be considered as a subclass of a structural health monitoring (SHM) system, which provides spatially-resolved information about loads (forces, moments, *etc*.) applied to a technical structure.

Multi-agent systems (MAS) can be used for a decentralized and self-organizing approach to data processing in a distributed system, like a sensor network (discussed in [1]), enabling information extraction, for example, based on pattern recognition [2], decomposing complex tasks in simpler cooperative agents. MAS-based data processing approaches can aid the material-integration of structural health monitoring applications, with agent processing platforms scaled to the microchip level, which offer material-integrated real-time sensor processing. The agent mobility, capable of crossing different execution platforms in mesh-like networks and agent interaction by using tuple-space databases and global signal propagation, aids with solving data distribution and synchronization issues in the design of distributed sensor networks, as already shown in [3,4].

In [5], the agent-based architecture considers sensors as devices used by an upper layer of controller agents. Agents are organized according to roles related to the different aspects to integrate, mainly sensor management, communication and data processing. This organization largely isolates and decouples the data management from changing networks, while encouraging the reuse of solutions.

Currently, there are only very few works related to low-resource agent processing platforms, especially related to sensor networks. Examples are presented in [6] and [7], but the proposed platform architectures do not match the constraints and requirements arising in multi-scale and multi-domain sensor networks. For example, in [8], a Java virtual machine (VM) approach is used, which is not scalable entirely to the hardware level and, therefore, limited to software-based designs.

The importance of the deployment of virtual machines in heterogeneous and multi-purpose sensor networks was already pointed out in [9]. In this work, a new operational paradigm for the programming and design of sensor network applications was addressed, showing the suitability of database-like communication approaches, which is proposed in a different way in this work using synchronized tuple-spaces for MAS. The system architecture also uses a stack-based bytecode interpreter with integer arithmetic, but supporting low-level instructions only (Java VM subset), though the VM can directly access sensors and network messages. There is no hardware implementation of the VM, degrading the performance significantly.

Usually, sensor networks are part of and connected to a larger heterogeneous computational network [5] and can be part of the emerging field of ambient intelligence, supporting intelligent behavior and information retrieval combined for ubiquitous computing (see [10] for details). The deployment of agents can overcome interface barriers arising between platforms differing considerably in computational and communication capabilities. That is why agent specification models and languages must be independent of the underlying run-time platform. The adaptive and learning behavior of MAS, central to the agent model, can aid in overcoming technical unreliability and limitations [11].

The capability of agents to migrate between different processing nodes (sensor node, computer, server, mobile device) extends the interaction domain and increases the capability to adapt to environmental changes, including the failure of network nodes [10]. Migration is closely related to the agent behavior, programming and architecture model, which immediately shifts the focus to the agent processing platform.

This work is based on an earlier data processing architecture described in [12] using virtual stack machines and mobile program code based on the FORTHinstruction set and which can migrate between different VMs and nodes of a distributed (sensor) network. A code morphing mechanism was used to enable self-modification of the program code at run-time. Code morphing is the capability of programs to modify their own code or the program code of other programs. This early approach matched only partially the agent model and had limited practically use due to very fine-grained code modification at the instruction word level. Furthermore, the VM architecture supported only coarse-grained parallelism. The first considerations to improve the early approach and to match it with a more reasonable agent model were presented in [13], and this is finally investigated, refined and evaluated in more depth in this work. The FORTH programming language (PL) combines the advantages of being a low-level machine and a structured programming language with statements, which can be directly executed by a VM interpreter [14].

In [15], a register-based virtual machine deployed in wireless sensor networks was proposed, arguing for the lower code size and higher processing speed of a register machine compared with stack machine code. However, a stack machine has the advantage of a simpler control and data processing unit interacting mostly with the top elements of the stacks, speeding up the code processing and simplifying the hardware design significantly [16]. Hence, processing speed, which means computational latency, does not only depends on the number of instructions to be processed. Furthermore, there are complex FORTH control flow instructions, like loops, which compensate for a higher number of instructions required for data processing. One major advantage of the FORTH instruction set is that most instructions carry no operand, which eases the code morphing performed by the virtual machine at run-time.

Commonly, programs are closely coupled with the interface of the execution platform (system data structures and functions). Furthermore, binary programs cannot exchange and share code in a simple way, and the issues with non-matching dynamic libraries are well known. The FORTH PL avoids this lack of code exchange and sharing by providing a simple dictionary approach. Programs can store new function words and retrieve functions by using textual string identifiers, enabling different programs to exchange and share program code, assuming the functions have no side effects (dependencies). FORTH programs can directly access the dictionary. This dictionary approach can be used for agents to share behavioral activities and utility functions. Furthermore, it supports self-organization. An overview of the FORTH programming language can be found in [17]. FORTH-based stack processors are well suited for massive parallel and distributed computing systems, referring, e.g., to [18].

The new processing platform architecture is optimized for the agent programming model and language used in this work. The FORTH programming language was extended with agent-specific actions (migration, forking, communication), supported entirely at the machine level.

**Figure 1.**
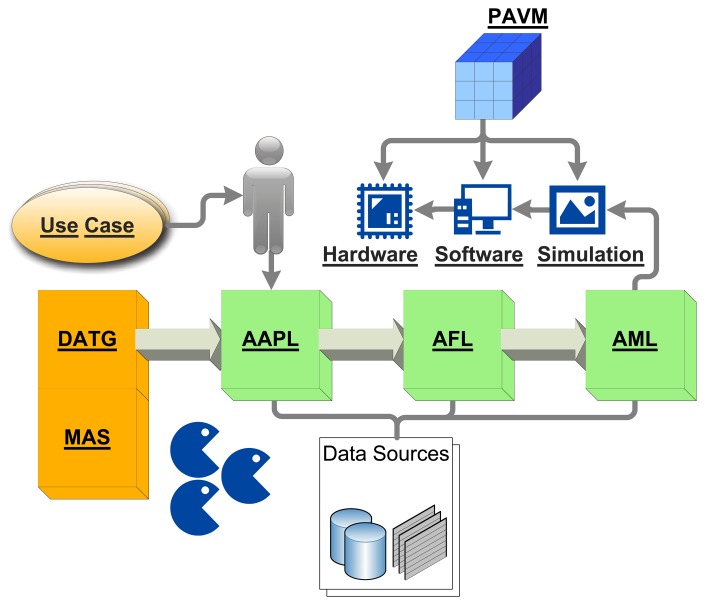
Design overview and design flow: from the model, to the programming, to the machine level, with one unique agent model (DATG, dynamic activity-transition graph; AAPL, Agent-orientated Programming Language; AFL, Agent ForthProgramming Language; AML, Agent Forth Machine Language; MAS, multi-agent system; PAVM, pipelined agent virtual machine).

Sections 2 and 3 give an overview of the front-end using the Activity-based and Agent-orientated Programming Language, AAPL, and the agent behavior model, leading to the Agent Forthand Agent Machine Language (AFL/AML), which is discussed in detail in Section 5. This section helps to understand the processing and morphing of agents having their origin in high-level behavior models with a machine, which can fit entirely on 50 mm^2^ silicon! The pipelined agent virtual machine (PAVM) processing architecture and its operational semantics is explained in detail in Section 4, followed by the design flow and transformation rules in Section 6. A simulation environment is introduced in Section 7, which performs a network and platform simulation using agent-based simulation techniques at a fine-grained architectural level. The simulation environment can be used to study the deployment of MAS in complex network environments. Furthermore, it can be connected to a real-world sensor network, too. Finally, the suitability of the proposed programming and processing model is demonstrated with an extended case study. Figure 1 summarizes all parts of this work and their relationships.

What is novel with respect to other approaches?

- Large-domain reactivity in heterogeneous networks is provided by mobile state-based agents capable of reconfiguring the agent behavior (activity-transition graph modification) for each particular agent at run-time, including the inheritance of (modified) agent behavior, which increases the reliability and autonomy of multi-agent systems.
- Agent interaction offered by a tuple-space database and global signal propagation aids with solving data distribution and synchronization issues in distributed systems design (machine-to-machine communication), whereby tuple spaces represent the knowledge of single and multiple agents.
- The common agent programming language, AAPL, and processing architecture, PAVM, enables the synthesis of standalone parallel hardware implementations or, alternatively, standalone software implementations and behavioral simulation models, compatible at the operational and processing level, which enables the design and testing of large-scale heterogeneous systems.
- An agent instantiation is represented by and the behavior is implemented with a unified, very compact code frame consisting of machine instructions with embedded (private) agent data and all control units, like relocation lookup tables and the transition network section. The code frame can migrate between nodes, preserving the control and data state of an agent.
- AAPL provides powerful statements for computation, agent control, agent interaction and mobility with static and limited resources.
- An intermediate and machine language, AFL/AML, is based on the stack machine FORTH programming language, matching AAPL well and which offers direct transformation of the AAPL behavior model and the AAPL statements to the machine VM level.
- A token-based pipelined multi-core stack VM architecture for the agent processing (PAVM), which is suitable for hardware microchip implementations on register-transfer level and system-on-a-chip architectures, offers optimized computational resources and exceptional speed, requiring less than 1-M gates. There are alternative efficient software implementations of the VM, fully coded and operationally compatible.
- The processing platform is a standalone unit, which does not require any operating system (OS) and boot code for initialization, leading to a low start-up time latency, which is well suited for self-powered devices. All agent-specific actions, like migration or communication, are implemented at the VM machine level.
- There is improved scaling in large heterogeneous network applications, due to low host platform and communication dependencies of the VM and the agent FORTH programming model.

## 2. Agent Behavior Modeling: The Activity-Based Agent Model and Graphs

The implementation of mobile multi-agent systems for resource-constrained embedded systems with a particular focus on the microchip level is a complex design challenge. High-level agent programming and behavior modeling languages can aid with solving this design issue. Activity-based agent models can aid with carrying out multi-agent systems on hardware platforms.

The behavior of an activity-based agent is characterized by an agent state, which is changed by activities. Activities perform perception, plan actions and execute actions modifying the control and data state of the agent. Activities and transitions between activities are represented by an activity-transition graph (ATG). The Activity-Based and Agent-orientated Programming Language, AAPL (detailed description in [16]), was designed to offer modeling of the agent behavior at the programming level, defining activities with procedural statements and transitions between activities with conditional expressions (predicates). Though the imperative programming model is quite simple and closer to a traditional PL, it can be used as a common source and intermediate representation for different agent processing platform implementations (hardware, software, simulation) by using a high-level synthesis approach.

### 2.1. Agent Classes

The agent behavior, perception, reasoning and the action on the environment are encapsulated in agent classes, with activities representing the control state of the agent reasoning engine and conditional transitions connecting and enabling activities. Activities provide a procedural agent processing by a sequential execution of imperative data processing and control statements. Agents can be instantiated from a specific class at run-time. A multi-agent system composed of different agent classes enables the factorization of an overall global task into sub-tasks, with the objective of decomposing the resolution of a large problem into agents in which they communicate and cooperate with one other.

The activity-graph based agent model is attractive due to the proximity to the finite-state machine model, which simplifies the hardware implementation.

**Figure 2.**
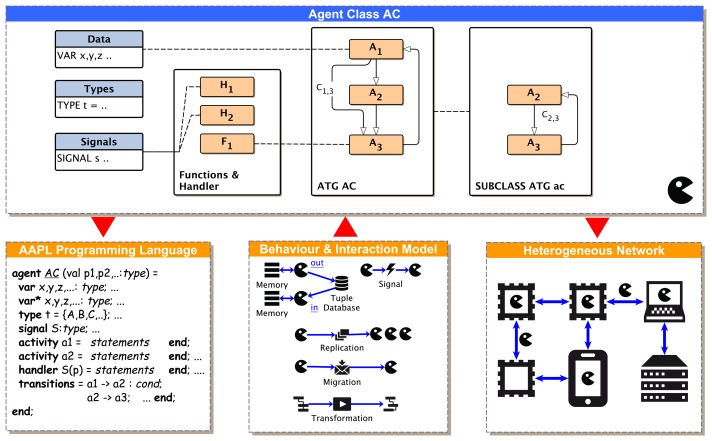
Agent behavior programming level with activities and transitions (Activity-Based and Agent-orientated Programming Language (AAPL) (**left**)); agent class model and activity-transition graphs (**top**); agent instantiation, processing and agent interaction on the network node level (**right**) [16].

An activity is started by a transition depending on the evaluation of (private) agent data (conditional transition) related to a part of the agents' belief in terms of the belief-desire-intention (BDI) architecture or started by unconditional transitions (providing sequential composition), shown in Figure 2. Each agent belongs to a specific parameterizable agent class, AC, specifying local agent data (only visible for the agent itself), types, signals, activities, signal handlers and transitions.

Definition: There is a multi-agent system (MAS) consisting of a set of individual agents {a_1_,a_2_,..}. There is a set of different agent behaviors, called classes, C = {AC_1_, AC_2_,..}. An agent belongs to one class. In a specific situation, an agent Ag*_i_* is bound to and processed on a network node N*_m,n_*_,.._ (e.g., a microchip, a computer or a virtual simulation node) at a unique spatial location (m,n). There is a set of different nodes, N = {N_1_, N_2_,..}, arranged in a mesh-like network with peer-to-peer neighbor connectivity (e.g., two-dimensional grid). Each node is capable of processing a number of agents n*_i_*(AC*_i_*) belonging to one agent behavior class AC*_i_* and supporting at least a subset of C′⊆C. An agent (or at least its state) can migrate to a neighbor node, where it continues working. Each agent class is specified by the tuple AC = 〈A,T,F,S,H,V〉. A is the set of activities (graph nodes); T is the set of transitions connecting activities (relations, graph edges); F is the set of computational functions; S is the set of signals; H is the set of signal handlers; and V is the set of body variables used by the agent class.

### 2.2. The Dynamic ATG and Sub-Classing

**Figure 3.**
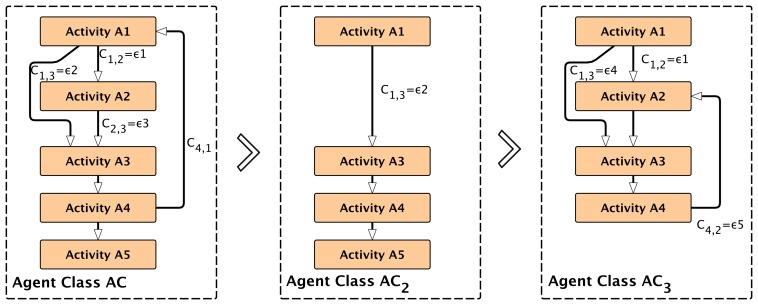
Dynamic activity-transition graph (ATG) transformation at run-time by modifying the set of transitions and activities, creating new agent (sub-)classes from an original root class.

Usually, agents are used to decompose complex tasks into simpler ones. Agents can change their behavior based on learning and environmental changes or by executing a particular sub-task with only a sub-set of the original agent behavior. The case study in Section 8. shows one example of a self-organizing multi-agent system with different agent behaviors and goals forked from one original root agent. An ATG describes the complete agent behavior. Any sub-graph and part of the ATG can be assigned to a subclass behavior of an agent. Therefore, modifying the set of activities *A*and transitions *T* of the original ATG introduces several sub-behaviors for implementing algorithms to satisfy a diversity of different goals. The reconfiguration of activities *A*′ = {*A*_1_⊆*A*,*A*_2_⊆*A*, ..} from the original set *A* and the modification or reconfiguration of transitions *T′* = {*T*_1_,*T*_2_ ,..} enable dynamic ATGs and agent sub-classing at run-time, shown in Figure 3.

## 3. Agent Behavior Programming: The High-Level AAPL

The AAPL (details can be found in [3]) offers statements for parameterized agent instantiation, like the parameterized creation of new agents and the forking of child agents inheriting the control and data state of the parent agent.

### 3.1. Agent Interaction and Coordination

Multi-agent and group interaction are offered with synchronized Linda-like tuple database space access operations and peer-to-peer interaction using signal propagation carrying simple data delivered to and processed by the signal handlers of agents. The tuple-space model, first introduced by the coordination language, Linda [19], is basically a shared memory database used for synchronized data exchange among a collection of individual agents, which was proposed in [20] and [8] as a suitable MAS interaction and coordination paradigm. Synchronization is offered by matching producer commitments of tuples and consumer requests for tuples. If a consumer requests a tuple that is not available, it will be blocked (waiting) until a producer commits a matching tuple, which is explained later.

A tuple database stores a set of n-ary data tuples, *t_n_* = (*v*_1_,*v*_2_,..,*v_n_*), an n-dimensional value tuple. The tuple space is organized and partitioned into sets of n-ary tuple sets ∇ = {*TS*_1_,*TS*_2_,..,*TS_n_*}. A tuple is identified by its dimension and the data type signature. Commonly, the first data element of a tuple is treated as a key. Agents can add new tuples (the output operation) and read or remove tuples (the input operations) based on the tuple pattern and pattern matching, *p_n_* = (*v*_1_,*p*_2_?, ..,*v_j_*,..,*p_i_*?,.,*v_n_*), a n-dimensional tuple with actual and formal parameters. Formal parameters are wildcard placeholders, which are replaced with values from a matching tuple. The input operations can suspend the agent processing if there is actually no matching tuple available. After a matching tuple is stored, blocked agents are resumed and can continue processing. The pattern of tuples matches iff the tuples have the same arity (equal to the number of elements), all actual values match and all formal parameters can be satisfied (e.g., the data type of actual values and formal parameters must be equal). Therefore, tuple databases provide inter-agent synchronization, too. This tuple-space approach can be used to build distributed data structures, and the atomic tuple operations provide data structure locking. The distributed tuple spaces represent the knowledge of agents and the history. The scope of a tuple-space is limited in this work to the node domain.

In contrast, signals, which can carry additional scalar data values, can be used for local (in terms of the node scope) and global (in terms of the network scope) domain agent interaction. In contrast to the anonymous tuple-space interaction, signals are directly addressed to a specific agent or a group of agents. The deliveries of signals are not reliable in the case that the agents raising and receiving the signal are not processed on the same node.

### 3.2. Agent Mobility

Agent mobility is offered by a simple move operation, which migrates the agent to a node in the neighborhood, assuming mesh-like networks, not necessarily with a static topology. Communication links are assumed as unreliable, which can be tested by an agent in advance.

### 3.3. Agent Classes

Agent classes are defined by their parameters, variables, activities and transition definitions, reflecting the ATG model. Optionally, an agent class can define additional functions for computation and signal handlers. There are several statements for ATG transformations and composition. Transitions and activities can be added, removed or changed at run-time.

Appendix A.1 introduces a short notation, which is a one-to-one and isomorphic mapping of the AAPL. This short notation is used in the following section and is used to describe the agent behavior in the case-study section.

**Figure 4.**
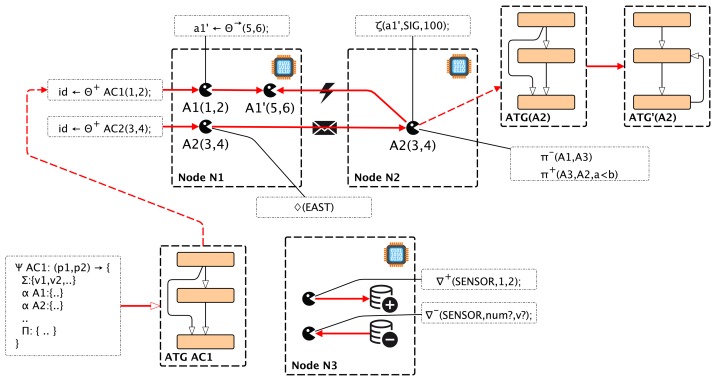
Effects of AAPL statements on the behavior of a multi-agent system.

Figure 4 shows the effects of selected major AAPL statements on the behavior of a mobile multi-agent system consisting of agents instantiated from different agent behavior classes.

## 4. Architecture: Agent Processing Platform

The requirements for the agent processing platform can be summarized as: (1) the suitability for microchip-level (SoC) implementations; (2) the support of a standalone platform without any operating system; (3) the efficient parallel processing of a large number of different agents; (4) the scalability regarding the number of agents processed concurrently; and (5) the capability for the creation, modification and migration of agents at run-time. The migration of agents requires the transfer of the data and the control state of the agent between different virtual machines (at different node locations). To simplify this operation, the agent behavior based on the activity-transition graph model is implemented with program code, which embeds the (private) agent data, as well as the activities, the transition network and the current control state. It can be handled as a self-contained execution unit. The execution of the program by a virtual machine (VM) is handled by a task. The program instruction set consists of zero-operand instructions, mainly operating on the stacks. The VM platform and the machine instruction set implement traditional operating system services, too, offering a full operational and autonomous platform, with a hybrid RISC and CISC architecture approach. No boot code is required at start-up time. The hardware implementation of the platform is capable of operating after a few clock cycles, which can be vital in autonomous sensor nodes with local energy supply from energy harvesting. An ASICtechnology platform requires about 500–1000-k gates (16-bit word size), and can be realized with a single SoC design.

### 4.1. Platform Architecture

**Figure 5.**
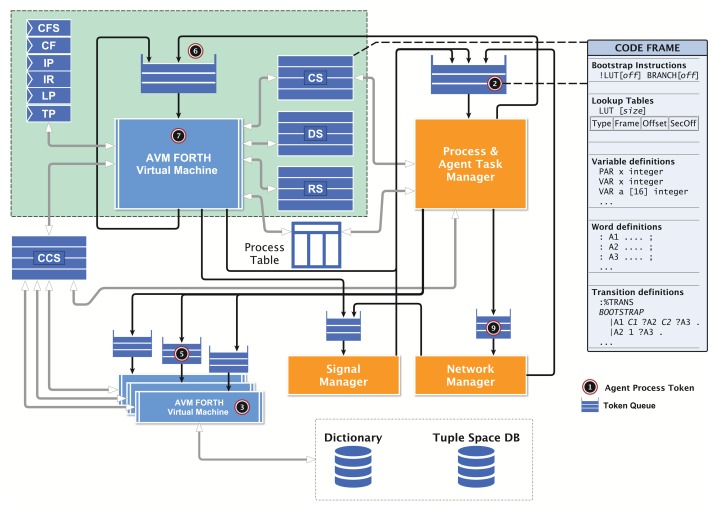
(**Left**) The agent processing architecture based on a pipelined stack machine processor approach. Tasks are execution units of the agent code, which are assigned to a token passed to the VM by using processing queues. The control state is stored in and restored from the process table. After execution, the task token is either passed back to the input processing queue or to another queue of either the agent manager or a different VM; (**Right**) The content and format of a code frame.

The virtual machine executing tasks is based on a traditional FORTH processor architecture and an extended zero-operand word instruction set (*α*FORTH), discussed in Section 5. Most instructions directly operate on the data stack *DS* and the control return stack *RS.* A code segment *CS* stores the program code with embedded data, shown in Figure 5. There is no separate data segment. Temporary data are stored only on the stacks. The program is mainly organized by a composition of words (functions). A word is executed by transferring the program control to the entry point in the *CS*; arguments and computation results are passed only by the stack(s). There are multiple virtual machines with each attached to (private) stack and code segments. There is one global code segment *CCS* storing global available functions and code templates, which can be accessed by all programs. A dictionary is used to resolve the *CCS* code addresses of global functions and templates. This multi-segment architecture ensures high-speed program execution, and the local *CS* can be implemented with (asynchronous) dual-port RAM (the other side is accessed by the agent manager, as discussed below) and the stacks with simple single-port RAM. The global *CCS* requires a Mutex scheduler to resolve competition by different VMs.

The register set of each VM consists of: (x0211B) = {*CF*, *CFS*, *IP*, *IR*, *TP*, *LP*, *A*, .. , *F* }. The code segment is partitioned into physical code frames. The current code frame that is processed is stored in the code frame pointer register (*CF*). The instruction pointer (*IP*) is the offset relative to the start of the current code frame. The instruction word register (*IR*) holds the current instruction. The look-up table pointer register *LP* stores an absolute code address pointing to the actual relocation LUT in the code frame, and the transition table pointer register *TP* stores an absolute address pointing to the currently used transition table (discussed later). The registers *A* to *F* are general purpose registers.

The program code frame (shown on the right of Figure 5) of an agent consists basically of four parts: (1) a lookup table and embedded agent body variable definitions; (2) word definitions defining agent activities, signal handlers (procedures without arguments and return values) and generic functions; (3) bootstrap instructions, which are responsible for setting up the agent in a new environment (*i.e.*, after migration or on the first run); and (4) the transition table calling activity words (defined above) and branching to succeeding activity transition rows, depending on the evaluation of conditional computations with private data (variables). The transition table section can be modified by the agent by using special instructions, explained in Section 5.4. Furthermore, new agents can be created by composing activities and transition tables from existing agent programs, creating subclasses of agent super classes with a reduced, but optimized, functionality. The program frame (referenced by the frame pointer *CF*) is stored in the local code segment of the VM executing the program task (using the instruction pointer, *IP*). The code frame loading and modifications of the code are performed by the virtual machine and the agent task manager only. A migration of the program code between different VMs requires a copy operation applied to the code frame. Code morphing can be applied to the currently executed code frame or to any other code frame of the VM, referenced by the shadow code frame register (*CFS*).

Each time a program task is executed, the stacks are initially empty. After returning from the current activity execution, the stacks are left empty, too. This approach enables the sharing of only one data and return stack by all program tasks executed on the VM to which they are bound! This design significantly reduces the required hardware resources. In the case of a program task interruption (process blocking) occurring within an activity word, the stack content is morphed to code instructions, which are stored in the boot section of the code frame, which is discussed later. After the process resumption, the stacks can be restored.

Each VM processor is connected to the agent process manager (PM). The VM and the agent manager share the same VM code segment and the process table (PT). The process table contains only basic information about processes required for the process execution. The column entries of a process table row are explained in Table 1.

### 4.2. Token-Based Agent Processing

Commonly, the number of agent tasks *N_A_* executed on a node is much larger than the number of available virtual machines *N_V_* . Thus, efficient and well-balanced multi-task scheduling is required to get the proper response times of individual agents. To provide fine-grained granularity of task scheduling, a token-based pipelined task processing architecture was chosen. A task of an agent program is assigned to a token holding the task identifier of the agent program to be executed. The token is stored in a queue and consumed by the virtual machine from the queue. After a (top-level) word is executed, leaving an empty data and return stack, the token is either passed back to the processing queue or to another queue (e.g., of the agent manager). Therefore, the return from an agent activity word execution (leaving empty stacks) is an appropriate task scheduling point for a different task waiting in the VM processing token queue. This task scheduling policy allows fair and low-latency multi-agent processing with fine-grained scheduling.

Tokens are colored by extending tokens with a type tag. There are generic processing tokens, signal processing tokens and data tokens, for example, appearing in compounds with signal processing tokens, which are discussed later.

Each VM interacts with the process and agent task manager. The process manager passes process tokens of ready processes to the token queue of the appropriate VM. Processes that are suspended (*i.e.*, waiting for an event) are passed back to the process manager by transferring the process token from the current VM to the manager token queue.

### 4.3. Instruction Format and Coding

The width of a code word is commonly equal to the data width of the machine. There are four different instruction code classes: (1) value; (2) short command; (3) long command Class A; and (4) long command Class B. A value word is coded by setting the most significant bit of the code word (MSB) and filling the remaining bits (N-1, N machine word size) with the value argument. To enable the full range of values (full data size N bit), a sign extension word can follow a value word setting the most significant bit. A short command has a fixed length of eight bits, independent of the machine word and data width. Short commands can be packed in one full-sized word, for example two commands in a 16-bit code word. This feature increases the code processing speed and decreases the length of a code frame significantly. The long commands provide N-4 (class A) and N-7 (class B) bits for argument values.

### 4.4. Process Scheduling and VM Assignment

The token-based approach enables fine-grained auto-scheduling of multiple agent processes already executed sequentially on one VM with a FIFO scheduling policy. A new process (not forked or created by a parent) must be assigned to a selected VM for execution. There are different VM selection algorithms available: round-robin, load-normalized, memory-normalized and random. The VM selection policy has a large impact on the probability of the failure of a process creation and process forking by a running process, requiring child agents to be created on the same VM!

## 5. Agent FORTH: The Intermediate and the Machine Language

The FORTH programming language corresponds to an intermediate programming language level, with constructs from high-level languages, like loops or branches, and low-level constructs used in machine languages, like stack manipulation. The *α*FORTH (AFL) instruction set *I_AFL_* consists of a generic FORTH sub-set *I_DF_* with common data processing words (summarized in Appendix A.2) operating on the data and a return stack used for computation, a control flow instruction set *I_CF_*, *i.e.*, loops and branches, a special instruction set *I_AP_* for agent processing and creation, mobility and agent behavior modification at run-time based on code morphing and, finally, an agent interaction sub-set *I_AI_* based on the tuple space database access and signals. The AFL language is still a high-level programming language close to AAPL, which can be used directly to program multi-agent systems. The PAVM agent processing platform will only support a machine language sub-set (AML) with a small set of special low-level instructions *I_AM_* for process control, so that *I_AML_* ⊂ (*I_AFL_* ∪ *I_AM_*), and with some notational differences. Several complex and high-level statements of *I_AFL_* are implemented with code sequences of simpler instructions from the *I_AML_* set, and some of them are introduced in Section 6.

The (current) AML instruction set consists of 92 instructions, most of them being common FORTH data processing instructions operating immediately on stack values, and 31 complex special instructions required for agent processing, communication and migration. The AML instruction set is not fixed and can be extended, which leads to the increased resource requirement and control complexity of the VM.

### 5.1. Program Code Frame

**Figure 6.**
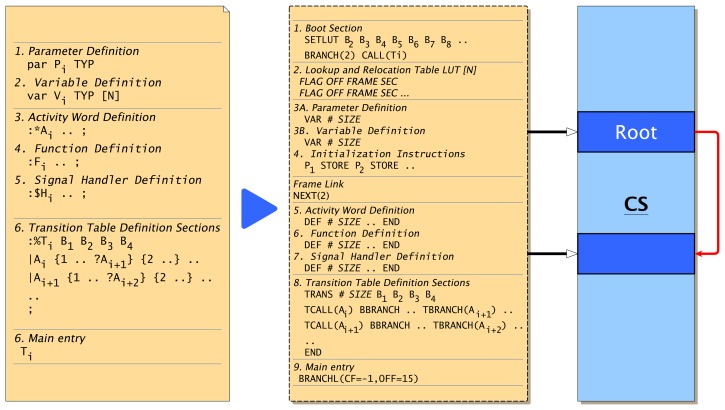
Logical code frame structure ((**left**) Agent Forth Language (AFL) source code) and (optionally split) physical code frame ((**middle**) Agent Machine Language (AML) machine code) with mapping to code partitions in the code segment (**right**).

An *α*FORTH code frame (see Figure 6) starts with a fixed sized boot section immediately followed by a program look-up relocation table (LUT). The instructions in the boot section are used to:

- set up the LUT offset register *LP* (always the first instruction),
- to enable program parameter loading (passed by the data stack),
- restore stack content after migration or program suspending and
- to branch the program flow to the transition table section.

The program counter *IP* points to the next instruction to be executed. The LUT is a reserved area in the program frame, which is initially empty, and is used by the VM to relocate the variable, word, transition table and transition table row references. A LUT row consists of the entries: {*Type*, *Code Offset*, *Code Frame*, *Secondary Offset*}. Possible row types are: *Type* = {FREE, PAR, VAR, ACT, FUN, FUNG, SIGH(S), TRANS}. The signal handler type SIGH is indeed a negative value, specifying the signal number S, which is related to the signal handler.

Within the program code, all address references from the frame objects, *i.e*., variables, user-defined words and transitions, are relocated by the LUT at run-time. This indirect addressing approach eases the reconfiguration of the program code at run-time and the code migration significantly. If a program frame is executed the first time or after a migration, the code frame is executed at the top level, where the LUT is updated and filled with entries by processing all object definitions of the frame from the beginning to the end. Code inside user-defined words is bypassed in this initialization phase. Variable, parameter and word definitions (var V, par P, :W) update at initialization-time entries in the LUT (code offset, code frame) and transition branches ?A update entries at run-time (the secondary offset specifying the relative offset of an activity call in the transition table section). At the programming AFL level, generic function, activity and signal handler word definitions are distinguished by different syntax (:F, :*A, :$S), whereas not at the machine instruction AML level (DEF).

After the LUT section, parameter and variable object definitions (private agent data) follow, and some top-level instructions are used to initialize agent parameters with values passed by the data stack.

The main part of the code frame consists of activity, function and signal handler word definitions (:F..; :*A..; :$S..; with the names F/A/S, respectively).

Finally, the transition table section is defined (:%T..;). A transition table consists of transition rows, which group all transitions outgoing from one specific activity, which is discussed later. Because more than one transition table can be defined, but only one may be used by a process at one time, a top-level transition table call is required at the end of the frame. A transition table contains a small boot section (four words) at the beginning, too. This boot section is used for the control of process resumption after a suspension, whereas the code frame boot section is used primarily to store data.

Beside pure procedural activity words (without any data passing, leaving the data stack unchanged), there are functional words passing arguments and results by using the data stack. Words not accessing private agent data can be exported (∷F) to a global dictionary (transferring the code to a *CCS* frame) and reused by other agents, which can import these functions (import W) referenced by their name, which creates a LUT entry pointing to the *CCS* code frame and offset relative to this frame. Global functions may not access any private agent data, due to the LUT-based memory relocation.

The code segment of a VM is divided into fixed sized partitions to avoid memory management with dynamic linked lists for free and used memory regions and memory fragmentation issues. A code frame always occupies a region of this fixed size code partition, the physical code frame, in the code segment of the respective virtual machine. Therefore, a single code frame is commonly limited to a minimum of 512 and a maximum of 2048 words of one code partition, depending on the VM implementation and *CS* overall size. In the case that an agent program does not fit in one code partition, physical code frames can be linked, forming one logical code frame, shown on the right side of Figure 6.

A physical code frame is specified by its address offset in the code segment, or by a partition index number (absolute index), or by a relative physical code frame number, relative to the first root frame of a process. The root frame always has the relative number one. This relative physical code frame numbering is required to support code frame migration between different VMs, where absolute code frame addresses and index numbers change, which is discussed later.

The last two words of the boot section are reserved and are used to control the code frame initialization and the current transition set table. Initially, they contain the {BRANCH(2),CALL(Ti)} word sequence. At the end of the code frame, there is a long branch to the last boot section word finally executing CALL(Ti). If the initialization of the code frame should be omitted, the BRANCH(2) word is replaced with a NOP operation by using code morphing, discussed in the following sections.

The following subsections introduce the special AFL/AML instruction set required for code morphing, agent interaction, agent creation and mobility. Most instructions get their arguments from the stacks and return results to the stacks. To illustrate the modification of stacks by instructions, a common stack notation is used: (a_1_a_2_a_3_ -- r_1_r_2_r_3_), showing the relevant top content of the stack before (left part) and after the instruction execution (right part), delimited by --. The top element of the stack is the right element (a_3_/r_3_ in this example). The return stack is prefixed with a R character.

### 5.2. Agent Processing

Agent processing involves the execution of activities and the computation and processing of transitions between activities based on private agent data (body variables). The transition computation is stored in the transition table words (:%TRANS..;). A transition table consists of transition rows, grouping all (conditional and unconditional) transitions for one outgoing activity. Each row starts with a activity word call |Ai, summarized in Table 2. After the return from the activity and a process schedule occur, a new activity transition is computed by evaluating Boolean expressions. The result of the computation is processed by a transition branch operation ?Aj, which branches to a different transition row (starting with |Aj) if the condition is true; otherwise, the next transition is evaluated. If currently no condition is satisfied and the end of a transition row is reached, the process is suspended, and the process token is passed back to the process manager. In this case, the process will be only resumed by the process manager if a signal was delivered to and processed by the process (e.g., an event occurred).

More than one transition table can exist and can be selected by using the !t statement. The reconfiguration of the transition table using the t+, t− and t* statements (add, delete, replace) requires code morphing capabilities, different from those instructions introduced in Section 5.4. The reconfiguration of the transition table, basically reduced to enabling and disabling of transitions at the machine level, is based on dynamic code blocks {n..}, which can be enabled or disabled using the blmod (BLMOD) instruction, explained in Table 4. The {*n..} block is enabled by default.

The generic program flow can be controlled using AFL and common FORTH branch and loop statements. At the machine level (AML), there are only three branch operations: (1) a conditional relative branch BRANCHZ (ΔIP), which redirects the program flow if the top of the data stack is zero; (2) an unconditional relative branch BRANCH (ΔIP); and (3) a long inter-frame branch BRANCHL(CF, IP).

### 5.3. Agent Creation and Destruction

New agents are created (or forked) by using a composition of the NEW, LOAD and RUN operations, discussed in Section 6. The suspend (SUSP) operation is usually inferred by the compiler in conjunction with other blocking instructions. An agent can be destroyed by using the kill operation. Table 3 summarizes these operations.

### 5.4. Agent Modification and Code Morphing

Code morphing is the capability of a program to modify its own code or the code of another program. Code morphing is used:

- to modify the boot section of a code frame and the boot section of a transition table,
- during process forking, migration and suspending to dump stack data into the boot section,
- to copy the variable, word and transition tables to a new code frame and
- to modify dynamic blocks, mainly used in the transition table, which enables or disables specific transitions.

Since data are embedded in the code frame of a process, code morphing is used here to modify data, too. Only twelve instructions supporting code morphing are required and are summarized in Table 4. There are explicit code morphing operations that can be used on the programming level, and there are implicit code morphing operations embedded in other control instructions, for example the QBLOCK and SUSP operations (see Table 2) used for modifying the code frame and transition table boot sections.

New code frames can be allocated using the new (NEW) instruction. The code frame can be allocated only from the VM of the current process. If the *init* argument is equal to one, then a default (empty) boot and LUT section is created, with sizes based on the current process. The code frame number *cf*# and the offset value *off* pointing to the next free code address in the morphing code frame is returned.

The load (LOAD) instruction has two purposes. Firstly, it can be used to load a code template from the global code segment CCS, which is resolved by the agent class number using the global dictionary. Secondly, it can be used to copy the current process code to the new code frame, which was already allocated by using the new command. If the template or the current process code spawns more than one frame, additional frames are allocated and linked.

New agents are created (or forked) by using a composition of the NEW, LOAD and RUN operations, discussed in Section 6.

Code can be modified by using the c> (TOC), v>c (VTOC), s>c (STOC) and r>c (RTOC) instructions. All of these operations are complex instructions with high operational power supported directly by the VM. Commonly, the code morphing instructions are used by the compiler for agent creation, forking and migration. Some process control instructions, like QBLOCK or SUSPEND, use code morphing implicitly to save the data and control state of the process in the boot sections.

All code morphing operations are applied to the code frame, which is referenced with the current *CFS* register, which can point to the current process frame or to any other code frame (limited to the code segment of the current process). The code morphing frame can be loaded by using the !cf (SETCF) operation. Basically, code morphing takes place by transferring code words from the data stack to a specified code offset position in the morphing frame by using the >c (TOC) operation. Code words (code snippets) can be pushed by using the c> (FROMC) operation, which copies code words following this word from the current process frame to the data stack. Value literal words can be created and transferred to the morphing frame with values stored on the data stack by using the v>c (VTOC) operation. The current entire stack contents can be dumped into the morphing frame by using the s>c (STOC) operation, excluding the arguments of this operation. Entire words from the current process can be copied to the morphing frame by using the r>c (RTOC) operation.

The transition table can be modified by using dynamic blocks and configurable block branches (BBRANCH), which can be enabled or disabled by using the BLMOD operation, inferred by high-level transition configuration functions t+, t− and t*, enabling and disabling transitions.

### 5.5. Tuple Database Space

The access of the database tuple space transfers n-ary data tuples to and reads or removes n-ary tuples from the database, based on pattern matching, which is part of the agent processing platform and directly supported at the machine level! Reading and removing of tuples is based on search pattern matching, consisting of actual parameter values and formal parameters replaced with values from matching tuples. The implementation of generic tuple space access, vital to the agent interaction model and heavily used, at the machine level is a challenge. Table 5 summarizes the AFL programming interface and AML subset, which reuses some instructions for efficiency. Tuple space operations (in and rd) can suspend the agent processing until a matching tuple is stored by another agent. This requires a special operational behavior of the machine instructions for further process management, which must save the control and data state (stack content) of this process in the frame and transition table boot sections.

The implementation complexity of the tuple space together with the code morphing operations is very high. Therefore, the high-level AFL input operations (in, tryin, rd, tryrd, ?exist, rm) are mapped on a reduced set of machine instructions {IN,RD} offering an enhanced platform resource sharing, selected by the t-parameter, explained in Table 5. The processing of the IN and RD operations by the platform VM profits from additional resource sharing in the VM.

### 5.6. Signal Processing

Signals ζ:S(*A*) carry simple information *A*, which is the (optional) argument of a signal. Signals are delivered to an appropriate signal handler of a specific agent, offering peer-to-peer agent communication. Signals are managed by the node signal manager. A signal *S* is delivered to an agent signal handler $S by inserting a signal processing token in the processing queue of the VM responsible for the parent process, followed by a signal and argument data token consumed by the VM immediately if the signal process is executed. The signal argument is pushed onto the data stack, and the signal handler word is called. After the return from the signal handler word, the signal processing token is converted into a wake-up event token and passed to the agent manager, which resumes the process in the case that the process waits for a signal event. On the one hand, using signal processing tokens and queues ensures that the parent agent process will not be preempted if executed with pending signals. On the other hand, signals can be process delayed, which is normally not critically, since the signal handler should modify only agent data used primarily for the transition decision process.

The raising of a signal from a source process passes an extended signal token to the signal manager, which either generates the above described signal processing token sequence, which is passed to the VM processing queue, or encapsulates the signal in a message, which is sent to a neighbor node by the network manager (sketched in Figure 5). A process migrating to a neighbor node leaves an entry in a process cache table, providing routing path information for message delivery to the migrated process.

The available AFL/AML instructions are summarized in Table 6.

### 5.7. Agent Mobility

An agent program can migrate to a different VM on a neighbor node by executing the move operation specifying the relative displacement to the current network node, shown in Table 7. A code migration is a complex instruction, which requires the dumping of the control and data state in the boot section of the code frame. The connection status for the link in a specified direction can be tested with the ?link operation. The migration is handled by the agent process and network managers and requires the encapsulation of the code frame(s) in a message container. The header of the container contains some persistent information about a process, like the process identifier number, the parent process identifier (if any) and the delta position vector. All remaining information is contained in the program code and is initialized by restarting the program on the new node and VM.

**Figure 7.**
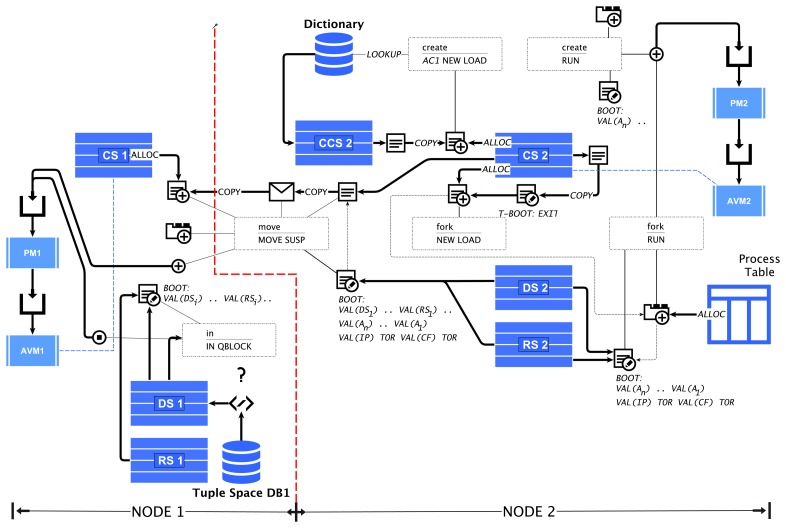
Effects of AML operations on code and stack memory, process management and token flow, shown partially for agents processed on two different connected platform nodes (Node 1, Node 2).

The effects of various important machine instructions are summarized and illustrated in Figure 7.

### 5.8. Examples

Take a look at the following very simple *α*FORTH code Example 1 implementing an agent performing a mean value computation of sensor values exceeding a threshold (agent parameter thr) with two body variables x and m, one agent class parameter thr, three activities {A_1_, A_2_, A_3_} and a transition network with some conditional transitions. The AAPL behavior model is shown on the right side. The sensor value will be read from the tuple space by using the ∇^−^/ in instruction in activity A_1_(tuple key ADC). The mean value is computed and stored in the database in activity A_2_. It is finally passed to the tuple database in activity A_3_ by using the ∇^+^/out instruction if the mean value exceeds a threshold. The agent is terminated after this action.

This code example requires 74 operational AML code words, and the total size of the code frame, including the boot section and the LUT created by the AFC compiler, is only 137 words. The AFL parameter definition appearing in Line 2 is treated like a variable definition, but with an additional parameter initialization added by the compiler following this definition immediately. After an agent process is instantiated from this program code, the entire program is executed at the top level and, therefore, initializing the parameter with values pushed to the data stack in the boot section, also added by the compiler. The last statement in the AFL program executes the transition network, starting the execution of the program (in this case, calling activity A1).



**Example 1** (**Left**) The code shows an *α* FORTH program derived from an AAPL-based agent behavior specification (**Right**) (in short notation).
*α*FORTH (AFL)              ⇐AAPL1enum TSKEY ADC SENSOR SENSOREV ;κ: {ADC, SENSOR, SENSOREV}2par thr integerψ mean_filter: thr → {3var x integer var m integer Σ: {x, m}4:A1 ADC 0bl0 2 in x ! ; α A_1_ : { ∇^−^ (ADC, x?) }5:A2 m @ x @ + 2 / m ! α A_2_ : { m← (m + x)/2;6  SENSOR m @ 2 out    ∇^+^(SENSOR, m) }7: A3 SENSOREV m @ 2 out $self kill; α A_3_ : { ∇^+^(SENSOREVENT, m); ⊗ ($self) }8:%trans Π : {9 |A1 m @ thr @ < ?A2 m @ thr @ >= ?A3 .  A_1_→A_2_ | m<thr10 |A2 1 ?A1 .  A_1_→A_3_ | m≥thr11 |A3 . ;  A_2_→A_1_ }12trans}


The reconfiguration of the ATG modifying the agent behavior using code morphing (see Example 2) enables agent sub-classing at run-time. This situation occurs in the employment of parent-child systems creating child agents getting an operationally-reduced subset from the parent agent. This approach has the advantage of high efficiency and performance due to the reduced code size. New agents can be created by simply forking an existing agent (fork), which creates a copy of the parent agent, including the data space. New agent programs (with different behavior) can be created by composing existing activities and by adding different transition tables. The capability to change an existing agent is limited to the modification of the transitions (enabling and disabling of dynamic blocks inside transition rows) and by removing activities. The transition table modification (and activity deletion) is the main tool for run-time adaptation of agents based on learning. The modified agent behavior can be inherited by forked child agents. In AFL/AML, customized agents can be assigned only a complete transition table that is already part of the current agent program.



**Example 2** Code morphing and agent creation related to the agent behavior modification.
αFORTH (AFL)⇐AAPL1t*(A1, 2)π*(A_1_ → A_2_ | x < y) -- replace all transitions A_1_->A_2_2t*(A1, 2)π+(A_1_ → A_2_ | x = 0) -- add transition A_1_->A_2_3100 1 fork a !a ← Θ^→^ (100); -- fork child agent4100 1 mean_filter create a !a ← Θ^+^ mean_filer(100); -- create new agent from class51 new !cf ref(A1) r>c ..a ← Θ^+^ (); -- create new customized agentref(T1) r>c ..α+ a (A1, A2, ..) π+a (A_1_ → A_2_ | x < y) ..‥100 1 run⨁a(100)


## 6. Synthesis and Transformation Rules

This section explains the mapping of the fundamental concepts of the ATG agent behavior and AAPL programming model and the transformation of the AFL program to AML machine code, primarily performed by the AFC compiler. The composition with only a small set of special AFL/AVMinstructions is capable of providing agent creation, forking, migration and modification by using code morphing, directly supported by the VM.

### 6.1. Agent Creation Using Code Morphing

New agent processes can be created by using code templates and the create statement, by forking the code and the state of a currently running process using the fork statement or by composing a new agent class from the current process.

Creating new and forking child processes is implemented with the previously introduced NEW, LOAD and RUN machine instruction sequences, defined in Equations (1) and (2), respectively.

(1) $$\frac{a_{1}\;a_{2}\;‥\;a_{n}\;n_{\text{args}}\;ac\;\text{create}}{\text{VAL}(0^{\text{noinit}})\;\text{NEW DUP TOR SWAP LOAD FROMR VAL}(0^{\text{new}})\;\text{RUN}}$$

(2) $$\frac{a_{1}\;a_{2}\;‥\;a_{n}\;n_{\text{args}}\;\text{fork}}{\text{VAL}(0^{\text{noinit}})\;\text{NEW DUP TOR VAL}(-1^{\text{fork}})\;\text{LOAD FROMR VAL}(1^{\text{fork}})\;\text{RUN}}$$

### 6.2. Agent Migration Using Code Morphing

Process migration requires the saving of the data and control state of the process in the frame and transition table boot sections. After migration, the code frame is fully reinitialized, including the loading of the process parameters. This requires the storage of the process parameter values on the data stack.

The migration is a two-stage process: the first stage is executed by the MOVE operation and the second by the SUSPEND operation, shown in Equation (3).

(3) $$\frac{dx\;dy\;\text{move}}{\begin{array}{l} \text{MOVE VAL}(-1^{\text{root}})\;\text{SETCF VAL}(1^{\text{codeoff}})\;\text{STOC TOR}\\ \text{REF}({\text{p}}_{1})\;\text{FETCH}‥\;\text{REF}({\text{p}}_{\text{n}})\;\text{FETCH}\\ \text{VAL}(\text{n})\;\text{FROMR VTOC VAL}(1^{\text{fullinit}})\;\text{SUSPEND} \end{array}}$$

**Example 3** (**Left**) The code shows an *α*FORTH program derived from an AAPL-based agent behavior specification (**Right**) (in short notation), posing the migration of agents.
αFORTH (AFL)                 ⇐AAPL1enum TSKEY ADC SENSOR ;κ: {ADC, SENSOR}2par dn integerψ mean_filter: dn → {3var x integer var dx integer var m integer Σ: {x, dx, m}4:A0 0 m ! 0 dx ! 0 dx ! ; α A_0_ : { m**←**0; dx← 0; }5:A1 ADC 0b10 2 rd x ! ; α A1 : { ∇^%^(ADC, x?) }6:A2 m @ x @ + 2 / m ! dx @ 1 + dx ! 1 0 move; α A_2_ : { m← (m + x)/2; dx←dx+1; ⇔ (EAST) }7:A3 SENSOR m @ 2 out self kill ; α A_3_ : { ∇^+^(SENSOR, m); ⊗($self) }8:%trans Π : {9 |A0 1 ?A1 .  A_0_→ A_1_10 |A1 dx @ dn @ < ?A2 dx @ dn @ = ?A3 .  A_1_→ A_2_ | dx<dn11 |A2 1 ?A3 .  A_1_→ A_3_ | dx=dn12 |A3 . ;  A_2_→ A_3_ }13trans}


In Example 3, a short AAPL program and the transformed corresponding AFL program is shown. The AAPL program implements a mobile agent traveling along the x-axis in the east direction in a mesh-like network using the ⇔/ move instruction (activity A_2_), sampling sensor values using the ∇^%^/ rd instruction reading the sensor value from the current node tuple space (activity A_1_) and computing the mean value of the sensor values. The agent class parameter dn determines the extension of the path in node hopping units. If the last node is reached, the computed mean value is stored in the local tuple database using the ∇^+^/ out instruction for further processing by other agents, performed in activity A_3_, which is started if the hop-counter dx is equal to dn (increased at each migration). The compiled AML code frame requires 181 words, including the embedded data space, boot section and LUT.

### 6.3. Code Frame Synthesis

The compiled AML machine program, which was synthesized from the previous Example 3, is shown with assembler mnemonics in the following Example 4. The program frame is partitioned according to Figure 6, beginning with a boot section (address range: 0–15), followed by the LUT (Start Address 16), and the parameter and variable definitions (Start Address 58) adding embedded data space after each object definition, which is part of the code frame. The LUT reserves four words for each object (variable, parameter, activity, function) used in this program frame. The object type (first column) is already filled.

The agent parameter is initialized by the store instruction at Address 74, getting the data from stack, which is pushed onto the data stack in the boot section (modified at agent process instantiation). For example, if the agent program is created with the parameter dn = 5, a typical boot section contains the instruction sequence SETLUT(18) VAL(5) .. BRANCH(2) CALL(9). The first instruction sets the LUT pointer relative to the code frame start, and the second pushes the argument onto the stack. The branch instruction ensures a complete initialization of the code frame (jumping over the transition section call). The last branch instruction (address range: 179–181) jumps back to the last instruction in the boot section calling the transition network.

The boot section is also used for the migration request, performed by the code in the address range 119–133. The move operation itself only prepares the migration (reset of the boot section), which is finalized by the last suspend instruction. The code between modifies the boot section by morphing the actual stack content to instructions in the boot section. After the migration, a full code frame initialization is required, therefore requiring the BRANCH (2) CALL (9) sequence at the end of the boot section. The boot section of the transition network (address range: 150–153) is modified for saving the current control state (that is, the instruction pointer) by creating a long branch to the next instruction to be executed after migration (within an activity word) and the full code frame initialization.

Each time a code frame is initialized by executing the top-level instructions, the LUT is updated by the VAR/DEF/TRANS instructions (updating the current code address). This self-initialization approach enables the modification of the code frame, e.g., reconfiguration and recomposition of agent programs.

The execution of the TCALL and TBRANCH instructions in the transition section relies on the LUT, too. The TBRANCH looks up and updates the secondary column (initially zero) of a LUT row for the relative address computation, reaching the respective TCALL. Again, this approach ensures the highest degree of flexibility and independence from any other computational unit or VM data.



**Example 4** Compiled AML assembler code from Example 3. First part: boot, LUT, variable and activity/function relocation section (*KIND NAME* [*off0 + off1*] #*LUT*); second part: machine instructions shown in *ADDR*: *AML* format.
BOOT[000000+0]LUTLUT [000016+2]PARdn [000058+3] #1VARx [000062+3] #2VARdx [000066+3] #3VARm [000070+3] #4WORDA0 [000076+3] #4WORDA1 [000089+3] #6WORDA2 [000101+3] #7WORDA3 [000134+3] #8TRANStrans [000147+3] #9*BOOT*0000 : SETLUT 180001 : NOP..0014 : BRANCH 20015 : CALL 9*LUT*0016 : LUT0017 : VAL 400018 : VAL 20019 : DATA0020 : DATA0021 : DATA *First LUT row*0022 : VAL 10023 : DATA0024 : DATA0025 : DATA *Second LUT row*..0058 : VAR0063 : VAL 10064 : VAL 10065 : DATA0062 : VAR0067 : VAL 20068 : VAL 10069 : DATA..*Parameter Initialization*0074 : REF 10075 : STORE*Activity A0*0076 : DEF0077 : VAL 50078 : VAL 100079 : VAL 00080 : REF 40081 : STORE0082 : VAL 00083 : REF 30084 : STORE0085 : VAL 00086 : REF 30087 : STORE0088 : EXIT*Activity A1*0089 : DEF0090 : VAL 60091 : VAL 90092 : VAL 10093 : VAL 20094 : VAL 20095 : VAL 00096 : IN0097 : QBLOCK0098 : REF 20099 : STORE0100 : EXIT*Activity A2*0101 : DEF0102 : VAL 70103 : VAL 330104 : REF 40105 : FETCH0106 : REF 20107 : FETCH0108 : ADD0109 : VAL 20110 : DIV0111 : REF 40112 : STORE0113 : REF 30114 : FETCH0115 : VAL 10116 : SUB0117 : REF 30118 : STORE0119 : VAL 10120 : VAL 00121 : MOVE0122 : VAL -10123 : SETCF0124 : VAL 10125 : STOC0126 : TOR0127 : REF 10128 : FETCH0129 : VAL 10130 : FROMR0131 : VTOC0132 : VAL 10133 : SUSP0134 : EXIT*Activity A3*0135 : DEF0136 : VAL 80137 : VAL 90138 : VAL 10139 : REF 40140 : FETCH0141 : VAL 20142 : VAL 00143 : OUT0144 : VAL -10145 : CLEAR0146 : EXIT*Transition Network Section*0147 : TRANS0148 : VAL 90149 : VAL 290150 : NOP0151 : NOP0152 : NOP0153 : NOP0154 : TCALL 50155 : VAL 10156 : TBRANCH 60157 : END0158 : TCALL 60159 : REF 30160 : FETCH0161 : REF 10162 : FETCH0163 : LT0164 : TBRANCH 70165 : REF 30166 : FETCH0167 : REF 10168 : FETCH0169 : GE0170 : TBRANCH 80171 : END0172 : TCALL 70173 : VAL 10174 : TBRANCH 80175 : END0176 : TCALL 80177 : END0178 : EXIT*Transition Section Call, referenced from Boot section*0179 : VAL 150180 : VAL -10181 : BRANCHL


## 7. Agent Platform Simulation

The proposed agent processing platform is a massive parallel data processing system. The composition of networks with these processing nodes creates a massive distributed system. The agent behavior model used in this work reflects the parallel and distributed system. However, it is a challenge to test and validate the operational and functional behavior of a MAS consisting of hundreds and thousands of agents processed on hundreds of agent platform nodes. The monitoring of such a large parallel and distributed system is nearly impossible in a technical real-world system. For this purpose, a multi-agent-based simulation environment is used to simulate the distributed agent platform network on architectural level. That means that all components, *i.e.*, the VM and the managers, shown in Figure 5 are simulated with non-mobile state-based agents and the SeSAm simulator [21], simulating the processing of code frames representing agents on the proposed platform architecture. This simulation model uses agents to simulate the processing of the agents introduced in Sections 2 and 3. In SeSAm, the agent behavior model is based on a similar, but simpler, ATG model compared with the AAPL model introduced in this work. SeSAm agents communicate with each other by accessing agent body variables of other agents (that is, a shared memory model). This approach is only suitable in a simulation environment and not in a real-world distributed deployment of agents.

Though the simulation model has no fixed timing model regarding the real processing platform (e.g., a microchip), a time step in the simulation is equivalent to the processing of one machine instruction, which corresponds roughly to 5–10 clock cycles required in an RTLimplementation of the PAVM platform for the same code processing. The relationship for a software implementation of the PAVM platform is about 100–1000 machine instructions on a generic microprocessor for each simulation step.

The simulation environment addresses two different simulation goals: (1) testing, profiling and validation of the agent processing platform; and (2) testing, profiling and validation of algorithms and multi-agent system use cases, for example event-based distributed sensor data processing in sensor networks. Technical failures, like connection losses or complete node failures, can be simulated using Monte-Carlo simulation methods.

**Figure 8.**
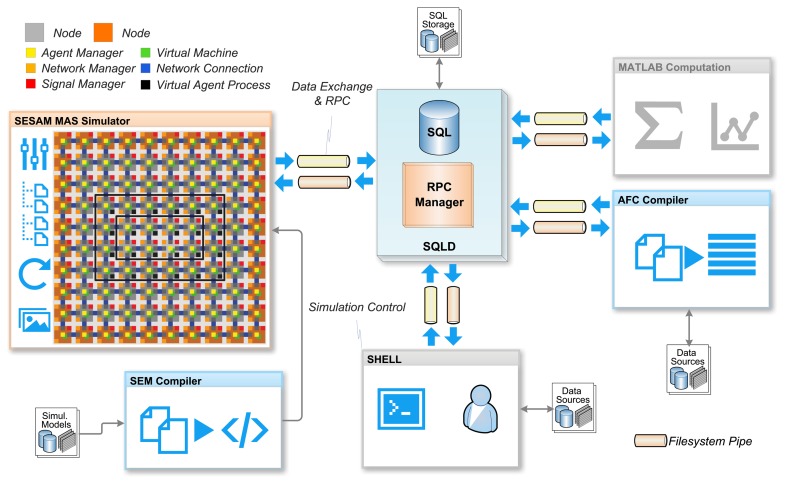
(**Left**) Simulation environment with the simulation world of a sensor network with sensor nodes containing the PAVMprocessing platform (with multiple VM and manager components, each simulated using an agent); (**Right**) The simulator operates on a database for storing output and reading input data (e.g., the program code).

The entire simulation environment uses a database for storing output and reading input data, e.g., the program code, shown in Figure 8. The SQLD database server not only provides a standard SQL-based database interface, it additionally provides an Remote Procedure Call (RPC) interface, which allows programs to communicate and synchronize with each other. This feature enables multi-domain simulations, for example incorporating external mathematical computations with MATLAB or FEM simulators for testing and evaluating structural monitoring systems (but, this work is not concerned with this). On the other hand, the code output of the AFL compiler AFC can be immediately stored in the database and read by the simulator.

The SeSAm simulator was originally only a GUI-based programming interface for the composition of the simulation model, which is not suitable for large models. To overcome this limitation, a textual representation of the SeSAm simulation model with the SEM language was developed, which can be compiled with the SEMC compiler to an XML simulation model, which can be imported directly by the simulator (native model file format).

The simulation world consists of a 10 by 10 mesh network of sensor nodes and some dedicated computational nodes at the outside of the network, which is not relevant for the following case study. Each network node consists of a process, signal and two network managers and four virtual processing machines, each with its own code and stack memory segments. The physical code frame size is set to 1024 words. Overall, 400 VMs with a total of seven million memory cells are simulated simultaneously. The simulator uses 1621 immobile (SeSAm) agents to simulate the platform components and the network. Furthermore, agents are used to simulate the network connections between nodes (resources in the terms of SeSAm). Each sensor node, represented by a node agent, provides a set of sensor values by storing data tuples in the node tuple database, which can be processed by other agents. There is a world agent, which updates the sensor values for all nodes. The set of sensor data is read from the SQL database, the dimension and the values of which depend on the use case being simulated.

## 8. Case Study: A Self-Organizing System

In this section, a self-organizing MAS (SoS) is implemented with AAPL and transformed to *α*FORTH to show the suitability and resource requirements of the proposed agent processing platform. The AFM machine code is tested and evaluated by using the agent-based platform simulation environment introduced in the previous section.

### 8.1. The Algorithms

Faulty or noisy sensors can disturb data processing algorithms significantly. It is necessary to isolate noise from well operating sensors. Usually, sensor values are correlated within a spatially close region, for example in a spatially-distributed load-measuring network using strain-gauge sensors. The goal of the following MAS is to find extended correlated regions of increased sensor intensity (compared to the neighborhood) due to mechanical distortion resulting from externally-applied load forces. A distributed, directed diffusion behavior and self-organization are used, derived from the image feature extraction approach (proposed originally by [22]). A single sporadic sensor activity not correlated with the surrounding neighborhood should be distinguished from an extended correlated region, which is the feature to be detected.

The feature detection is performed by the mobile exploration agent, which supports two main different behaviors: diffusion and reproduction. The diffusion behavior is used to move into a region, mainly limited by the lifetime of the agent, and to detect the feature; here, the region with increased mechanical distortion (more precisely, the edge of such an area). The detection of the feature enables the reproduction behavior, which induces the agent to stay at the current node, setting a feature marking and sending out more exploration agents in the neighborhood. The local stimulus *H*(*i*,*j*) for an exploration agent to stay at a specific node with coordinates (*i*,*j*) is given by Equation (4).

(4) $$\begin{array}{l} H(i,\;j)=\overset{R}{\underset{s=-R}{\text{∑}}}\overset{R}{\underset{t=-R}{\text{∑}}}\left\{\left\|S(i+s,j+t)-S(i,j)\right\|\leq \delta \right\}\\ S:\text{Sensor Signal Strength}\\ R:\text{Square Region around}(\text{i},\text{j}) \end{array}$$

The calculation of *H* at the current location (*i*,*j*) of the agent requires the sensor values within the square area (the region of interest (ROI)) *R* around this location. If a sensor value *S*(*i* + *s*,*j* + *t*) with *i, j*∈{− *R*,..,*R*} is similar to the value *S* at the current position (difference is smaller than the parameter *δ*), *H* is incremented by one.

If the *H* value is within a parameterized interval Δ= [*ϵ*_0,_*ϵ*_1_], the exploration agent has detected the feature and will stay at the current node to reproduce new exploration agents sent to the neighborhood. If *H* is outside this interval, the agent will migrate to a different neighbor node and restart the exploration (diffusion).

The calculation of *H* is performed by a distributed calculation of partial sum terms by sending out child explorer agents to the neighborhood, which can send out more agents until the boundary of the region *R* is reached. Each child agent returns to its origin node and hands over the partial sum term to his parent agent. Because a node in the region *R* can be visited by more than one child agent, the first agent reaching a node sets a marking MARK. If another agent finds this marking, it will immediately return to the parent. This multi-path visiting has the advantage of an increased probability of reaching nodes having missing (non-operating) communication links. An event agent, created by a sensing agent, finally delivers sensor values to computational nodes, which is not considered here.

The AAPL algorithm in short notation is shown in Algorithm 1, exhibiting a subclass definition for the neighborhood perceptor agents (helpers). The definition of the agent class *Explorer* begins at Line 6, defining two parameters *dir* and *radius*. The direction parameter determines the (initial) spatial migration direction of the agent, and the radius parameter limits the exploration region. The root class defines the activities {*init*, *percept*, *reproduce*, *diffuse*}, and the subclass, beginning at Line 78, defines the activities {*percept_neighbor*, *move*, *goback*, *deliver*}. Two signal handlers are installed (Lines 58, 61), processing *WAKEUP* and *TIMEOUT* signals. The main transition set of the root class (Π) is defined at Line 68, and the transition set of the child class exploring the neighborhood (π) is defined at Line 105.

The corresponding AFL program consists of 236 source code lines only and is compiled into 721 code and 197 data words with a total code frame size of 918 words. The explorer child subclass requires 648 code and data words (resulting in a 30% reduction of the code frame size).



**Algorithm 1** Definition of the explorer agent behavior class, including the explorer child subclass
1κ: { SENSORVALUE, FEATURE, H, MARK } *set of key symbols*2ξ: { TIMEOUT, WAKEUP } *set of signals*3δ: { NORTH, SOUTH, WEST, EAST, ORIGIN } *set of directions*4ε1 =3; ε2 = 6; MAXLIVE = 1; *some constant parameters*56Ψ Explorer: (dir,radius) → {7 * Body Variables *8 Σ: { dx, dy, live, h, s0, backdir, group } *global persistent variables*9 σ: { enoughinput, again, die, back, s, v } *local temporary variables*1011 *Activities*12 *α* init: {13  dx ← 0; dy ← 0; h ← 0; die ← false; group ← ℛ{0..10000};14  if dir ≠ ORIGIN then15   ⇔dir; backdir ← ϖ(dir)16  else17   live ← MAx0LIVE; backdir ← ORIGIN18   ∇^+^(H, $self, 0);19   ∇^%^(SENSORVALUE, s0?)20 }21 α percept: {22  enoughinput ← 0;23   ∀{nextdir∈δ | nextdir ≠ backdir ∧ ? Λ (nextdir)} do24   enoughinput++;25   Θ^→^ Explorer.child (nextdir,radius)26  τ^+^(ATMO, TIMEOUT)27 }28 α reproduce: {29  live--;30   ∇^x^(H, $self,?);31  if ?∇(FEATURE,?) then ∇**^−^**(FEATURE, n?) else n ← 0;32   ∇^+^(FEATURE,n+1);33  if live > 0 then34   π*(reproduce → init)35    ∀{nextdir∈δ | nextdir ≠ backdir ∧ ? Λ (nextdir)} do36    Θ^→^ (nextdir,radius)37   π*(reproduce → exit)38 }39 α diffuse: {40  live--;41  ∇^x^(H, $self,?);42  if live > 0 then43   dir ← ℛ{nextdir∈δ | nextdir ≠ backdir ∧ ?Λ (nextdir)}44  else45   die ← true46 }47 α exit: {⊗($self) }4849 inbound: (nextdir) → {50  case nextdir of51  | NORTH → dy > -radius52  | SOUTH → dy < radius53  | WEST → dx > -radius54  | EAST → dx < radius55 }5657 *Signal handler*58 ξ, TIMEOUT: {59  enoughinput ← 060 }61 ξ, WAKEUP: {62  enoughinput--;63  if ?∇(H, $self,?) then ∇^−^(H, $self,h?);64  if enoughinput < 1 then τ^−^(TIMEOUT);65 }6667 *Main Transitions*68 Π: {69  entry → init70  init → percept71  percept → reproduce | (h ≥ ε1 ∧ h ≤ ε2) ∧ (enoughinput < 1)72  percept → diffuse (h < ε1 ∨ h > ε2) ∧ (enoughinput < 1)73  reproduce → exit74  diffuse → init | die = false75  diffuse → exit | die = true76 }77 *Explorer child subclass*78 φ child: {79  α exit *imported from root class*80  ξ TIMEOUT81  ξ WAKEUP82  α percept_neighbour {83   if not ?∇(MARK, group) then84    back ← false; enoughinput ← 0; ∇^τ^ (MTMO, MARK, group); ∇^%^(SENSORVALUE, s?);85    h ← (if |s-s0| ≤ DELTA then 1 else 0);86     ∇^+^(H, $self, h);87    π*(percept_neighbour → move)88     ∀{nextdir∈δ | nextdir ≠ backdir ∧ ?Λ (nextdir) ∧ inbound(nextdir)} do89     Θ^→^ (nextdir, radius)90    π*(percept_neighbour → goback | enoughinput < 1)91    τ^+^(ATMO, TIMEOUT)92 }93 α move: {94  backdir ← ϖ(dir); (dx, dy) ← (dx, dy) + ∂(dir);95  ⇔dir;96 }97 α goback: {98  if ?∇(H, $self,?) then ∇^−^(MARK, $self, h?) else h ← 0;99  ⇔backdir;100 }101 α deliver: {102   ∇^−^(H, $parent, v?); ∇^+^(H, $parent, v+h);103  ξWAKEUP ⇒ $parent;104 }105 π: {106  entry → move107  move → percept_neighbour108  deliver → exit109  goback → deliver110  }111 }112}


**Figure 9.**
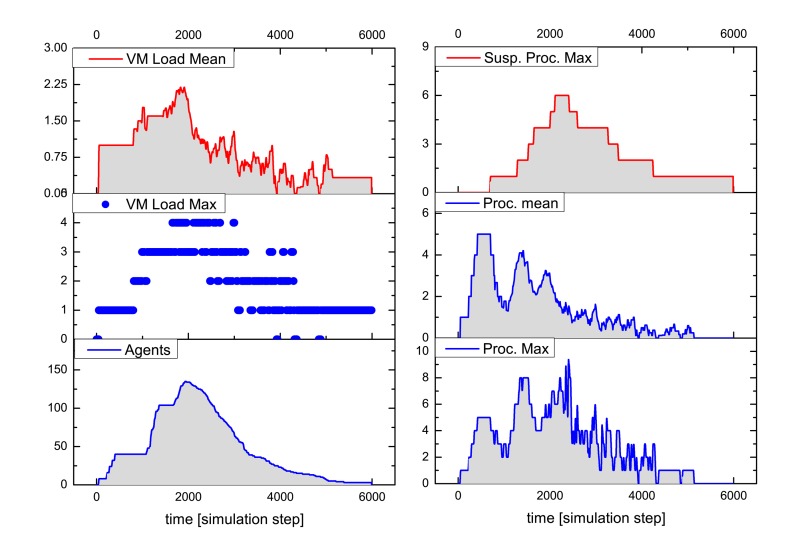
Analysis results for a typical run of the SoS multi-agent system (MAS) with a correlated cluster of 4 × 2 nodes having significant different sensor values compared with the neighborhood (with four VMs/node, the max and mean computation related to nodes in the region of active nodes processing at least one program/agent) (load: the fraction of processing to the idle time of a VM set).

**Figure 10.**
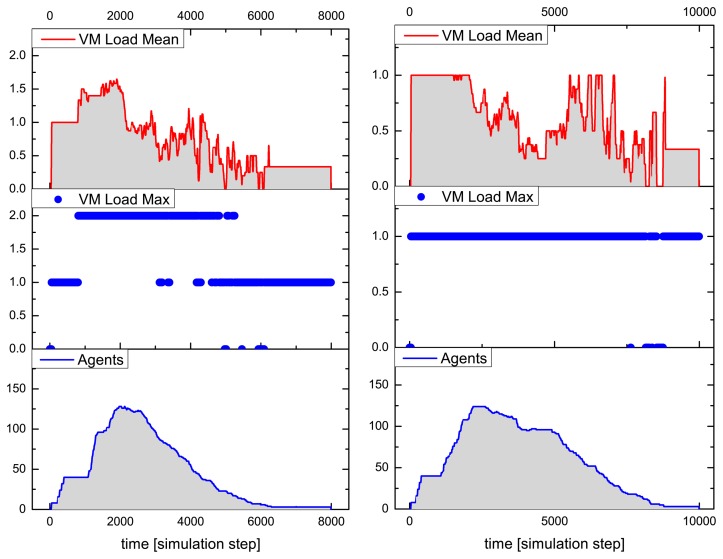
Analysis results with two VMs (**left**) and only one VM (**right**) per node (the feature is recognized after 6200 and 8800 simulation steps, respectively) (load: the fraction of processing to the idle time of a VM set).

Figures 9 and 10 summarize the analysis results for a typical simulation run of the above-described SoS MAS, with a stimulated sensor network region of four by two nodes having sensor values differing significantly from the neighborhood (shown in the inner black rectangle in Figure 8). The analysis shows the VM load factor, the agent processing statistics and the agent population (related to the SoS MAS) in the entire network for the test run. The VM load is the fraction of processing to the idle time of a VM, for each VM in the range [0.0,1.0], and is cumulated for all VMs of a node (node VM load factor). To clarify this, if all VMs are busy 100% of the time, the node load factor is *x* if the number of VMs per node is *x*. The mean value is an averaged node VM load factor of all nodes, which are processing SoS agents, and the maximum value is the peak value of one VM of this group.

The MAS population has a peak number of 140 agents, with originally eight root agents created by the sensor nodes. The analysis evaluates the temporally resolved processing load of the VMs in the extended region populated with explorer and explorer child agents only (in the outer black rectangle in Figure 8). Different platform configurations were investigated with four, two and one VM(s) per node. Depending on the number of VMs per node, the feature is recognized by the MAS after 5050, 6200 and 8800 simulation steps, respectively, which shows a significant performance decrease by reducing the available number of VMs. The average speedup compared with one VM for this specific MAS and network situation is 2.2 for four and 1.5 for two parallel processing VMs. The fine-grained platform simulation of program code processing requires only 100-times more simulation steps than a comparable pure behavior-based agent simulation (directly implementing AAPL agents with SeSAm agents, shown in [4]). Each explorer agent requires about 1000 machine instruction to achieve its goal and termination. Neglecting communication and migration time, the total computational execution time of an explorer agent on a hardware platform with a 10-MHz clock frequency requires less than 1 ms! A node in the populated region processes up to 10 different agent programs, shown in the right diagram of Figure 9.

## 9. Discussion and Conclusions

In this work, a novel agent processing platform architecture for code-based mobile agents in large-scale heterogeneous sensor networks, including low-resource microchip nodes, was introduced. The standalone agent processing platform, a multi-core stack processor, can be implemented entirely at the microchip level and requires no operating system and no boot code. Alternatively, the processing platform can be implemented efficiently in software with code and operational compatibility, enabling deployment in heterogeneous network environments, inter-connecting hardware and software platforms executed on generic microprocessors.

The stack-based FORTH programming language was extended with powerful statements for agent control, migration, replication and communication, available entirely at the machine level, resulting in the AFL programming language, comparable in expressiveness to the high-level agent programming language, AAPL, but focused on and optimized for the FORTH VM platform. The machine instruction set AML is a subset of the AFL programming language with agent process control extensions.

The introduced simulation environment simulates the processing of the program code and addresses two different simulation goals: (1) testing, profiling and validation of the agent processing platform; and (2) testing, profiling and validation of algorithms and multi-agent system use cases, for example event-based distributed sensor data processing in sensor networks. Technical failures, like connection losses or complete node failures, can be simulated using Monte-Carlo simulation methods.

### 9.1. Suitability

The proposed mobile program-based agent approach is well suited for massive distributed multi-agent systems with a common cooperation goal and subclassification features. Examples are self-organizing systems used for pattern and feature recognition or event-based sensor distribution in large-scale networks. These distributed algorithms require replication and diffusion behavior with neighborhood exploration by forked child agents delivering pre-computed information parts (divide and conquer strategy). The agent mobility, which supports the migration between different execution platforms in mesh-like heterogeneous networks, and the agent interaction by using tuple-space databases and global signal propagation aid with solving data distribution and synchronization issues in the design of distributed heterogeneous sensor networks. The AFL and AML program code can be efficiently compiled from a high-level agent behavior specification using the AAPL programming language. The agent processing VM supporting the AML instruction set was matched to and optimized for the AAPL behavior model and agent-specific statements. A typical program code size of an agent employed in sensor networks for sensor pre-processing and distribution consists of about 1000 words (assuming a 16-bit machine requiring only 2000 bytes). Due to powerful and expressive agent behavior-specific statements, the overall efficiency (regarding code size and processing speed) is considerably good, especially compared with generic register-based or Java VMs (for example, pointed out in [15] and [8]). The migration of agents requires only the transfer of the program code encapsulated in messages. A migrated program code frame can be started on the new node or VM, immediately leading to short start-up latencies. The code frame is self-initializing, which means there is no operating system service required.

### 9.2. Efficiency

The multi-core hardware implementation of the VM benefits significantly from the parallel processing of agents on different VMs. The software implementation can benefit from parallelization and pipelining by using a multi-threaded implementation of the VM architecture. The hardware VM requires about two to four clock cycles to execute simple instructions (see [16]) and about 10 to 100 clock cycles for complex statements like the SUSPEND or the IN/OUT operations. The software implementation of the VM requires about 100 host machine instructions to execute simple and about 1000–10,000 machine instructions to execute complex statements. The memory requirements of the hardware and software VM implementations depend on the maximal number of agents to be processed.

### 9.3. Drawbacks and Issues

The fixed-size code frame approach with embedded data is not suitable for large data sets, which must be carried by agents, for example a multi-dimensional matrix, like images.

Agent programs must be forked and created on the VM currently processing the parent process. If there are not enough physical code frames available for this particular VM, this operation fails. Load balancing based on consumed computation time and allocated frames can be required by using code frame migration from one VM to another VM on the same network node.

Task scheduling only occurs if the currently processed agent program returns from an activity word call. Activities with high computation time can delay the processing of other waiting agent programs significantly. Preemptive scheduling of tasks with time slice scheduling is required, which is actually not implemented.

### 9.4. Outlook

The low-resource requirement of the PAVM architecture enables the implementation of the agent processing platform with embedded and interpreted programming languages, e.g., JavaScript. A JavaScript implementation of the processing platform offers the merging of web services with sensor networks, extending the activity domain of agents significantly and enabling unified service-orientated sensing systems.

## Figures and Tables

**Table 1.** Process table (PT) row format and description.

**STATE**	**VM#**	**CFROOT**	**CFCUR**	**IP**	**ID**	**PAR**	**AWAIT**	**AWARG**	**POS**
Process state	Virtual Machine Number	Root code frame number of the process	Current code frame number of the process	Last/Next IP offset	Process identifier number	ID of the parent process	The reason for waiting	Await argument (key)	Delta position of the migrated process

**Table 2.** AFL/AML agent processing control instructions.

**AFL**	**AML**	**Stack**	**Description**
|Ai	TCALL(#)	(--) R(-- ip cf#)	Call next activity word A_i_. The word address offset and the code frame are taken from the LUT. The current code location (a call frame) is stored on the return stack. Only relative frame numbers may be used in call frames to enable process migration.
?Ai	TBRANCH(#)	(flag --)	Branch to the next transition row for start activity *A_i_* if the flag is true. The relative branch displacement for the appropriate TCALL(#) target is first searched by using the LUT entry for the respective activity (Sec. off. col.). If this fails, the entire transition section is searched (and the result is cached in the LUT).
{*n.. } {n.. }	BBRANCH(Δ)	( -- )	Dynamic block environment: a conditional branch that can be enabled (Δ > 0, block disabled) or disabled (Δ < 0, block enabled) using the BLMOD operation. If the branch is enabled, the block spawned by Δ is skipped.
.	END	(--)	End marker, which marks the end of a transition table row. The process is suspended if reached in the transition section.
?block	QBLOCK	(flag --)	Suspend code processing and save the stacks if the flag is not zero. If a schedule occurs, the current data and return stack content must be transferred and morphed to the boot code section with a branch to the current *IP* − 1, repeating the previous code word execution after process resumption.
suspend	SUSPEND	(.. flag --) R(.. --)	Suspend the execution. The current *CF* and *IP* + 1 are saved in the current transition table boot section with a long branch. If *flag* = 1, then the code frame is fully re-initialized after resumption, and the stacks must be already dumped into the boot section. If *flag* = −1, then the boot section is initialized, and the stacks are dumped; after resumption, the next instruction is directly executed without the full code frame setup by jumping directly to the transition table boot section.

**Table 3.** AFL/AML agent creation and destruction instructions.

**AFL**	**AML**	**Stack**	**Description**
fork	-	(arg1 .. argn #args -- pid)	Fork a child process. The child process leaves the current activity word immediately after forking, and the parent process continues after the fork operation.
create	-	(arg1 .. argn #args #ac -- pid)	Create a new agent process loaded from the agent class code template #*ac*.
-	RUN	(arg1 .. #args cf# flag -- id)	Start a new process with code frame (from this VM); returns the identifier of the newly-created process. The arguments for the new process are stored in the boot section in the code frame of the new process. If *flag* = 1, then a forked process is started. The boot section of the new code frame and the boot section of the transition table will be modified.
kill	pid = self: CLEAR EXIT	(pid --)	Terminate and destroy an agent. For self-destruction, the aid must be equal to −1. Executing an exit operation with an empty stack (clear) terminates an agent.

**Table 4.** AFL/AML code morphing words (off, code offset relative to the code segment start; cf#, code frame number; ref#, LUT object reference number).

**AFL**	**AML**	**Stack**	**Description**
new	NEWCF	(init -- off*^init^*^=1^cf#)	Allocates a new code frame (from this VM) and returns the code frame number. If *init* = 1, then a default boot and LUT section is generated, and the code offset is returned additionally.
load	LOAD	(cf# ac# --)	Load the code template of agent class acin the specified code frame number or make a copy of the current code frame (*ac* = −1).
c>	FROMC	(n -- c)	Push the *n*following code words on the data stack.
v>c	VTOC	(v n off -- off')	Convert *n* values from the data stack in a literal code word and extension if required. The new code offset after the last inserted word is returned.
>c	TOC	( c1 c2 .. n off --)	Pop *n* code words from the data stack, and store them in the morphing code frame starting at offset *off*.
s>c	STOC	(.. off -- off') R( .. --)	Convert all data, return stack values to code values and store them in the morphing code frame starting at offset *off*. Return the new offset after the code sequence.
r>c	RTOC	(off ref# -- off')	Transfer the referenced object (word, transition, variable) from the current process to the morphing code frame starting at offset *off*. Returns the new offset after the code sequence.
!cf	SETCF	(cf# --)	Switch the code morphing engine to the new code frame (number). The root frame of the current process can be selected with #*cf* = −1.
@cf	GETCF	(-- cf#)	Get the current code frame number (in CSfrom this VM).
t+ (A*_i_*, b#) t−(A*_i_*, b#) t* (A*_i_*, b#) !t (T*_i_*)	BLMOD TRSET	(ref# b# v sel --) (ref# --)	Modify the transition table, which can be selected by the !t statement. Each transition bound to an outgoing activity is grouped in a dynamic block environment. The transition modifiers reference the block number in the respective transition row. The t-operations are reduced to the AML operation BLMOD (modify a dynamic block). BLMOD can be used for global dynamic blocks, too (*sel* = 1: transition; *sel* = 0: activity; *sel* = −1: top-level).

**Table 5.** AFL/AML tuple data space access operations.

**AFL**	**AML**	**Stack**	**Description**
out	0 OUT	(a1 a2 .. d --)	Store a d-ary tuple (*a1*,*a2*,..) in the database.
mark	OUT	(a1 a2 .. d t --)	Store a d-ary temporary marking tuple in the database (after time-out *t*, the tuple is deleted automatically).
in	0 IN	(a1 a3 .. p d -- pi.. p2 )	Read and remove or read only a tuple from the database. Only parameters are returned.
rd	0 RD		
	QBLOCK		To distinguish actual and formal parameters, a pattern mask *p* is used (n-th bit = 1: the n-th tuple element is a value; n-th bit = 0: it is a parameter and not pushed on the stack).
tryin	IN	(a1 a3 .. p d t -- pi.. p2 0 )	Try to read and remove or read only a tuple. The parameter *t* specifies a time-out. If *t* = −1, then the operation is non-blocking. If *t* = 0, then the behavior is equal to the rd operation. If there is no matching tuple, the original pattern is returned with a status of one on the top of the data stack, which can be used by a following ?block statement. Otherwise a status of zero is returned and the consumed tuple. Only parameters are returned.
tryrd	RD	(a1 a3 .. p d t -- a1 a3 .. p d t 1)	
rm	−2 IN	(a1 a2 .. p d -)	Remove tuples matching the pattern. This is processed with a IN operation and *t* = −2.
?exist	−2 RD	(a1 a3 .. p d -0|1)	Check for the availability of a tuple. This returns one if the tuple does exist, otherwise zero. It is processed with a RD operation and *t* = −2.

**Table 6.** AFL/AML signal processing instructions.

**AFL**	**AML**	**Stack**	**Description**
signal S	-	(--)	Definition of a signal S.
: $S .. ;	DEF	(arg --)	Definition of a handler for signal S. The signal argument is pushed on the top of the data stack.
raise	RAISE	(arg sig# pid --)	Send a signal *S* with an argument to the process *pid*.
timer	TIMER	(sig# tmo --)	Install a timer (*tmo* > 0) raising signal *sig* if the time-out has passed. If *tmo* = 0, then the timer is removed.

**Table 7.** AFL/AML agent mobility instructions.

**AFL**	**AML**	**Stack**	**Description**
move	MOVE	(dx dy --)	Migrate the agent code to the neighbor node in the given direction. The current data and return stack content are transferred and morphed into the boot code section. The transition boot section is loaded with a branch to the current *IP* + 1.
?link	LINK	(dx dy -- flag)	Check the link connection status for the given direction. If *flag* = 0, then there is no connection; if *flag* = 1, then the connection is alive.

**Figure A1.**
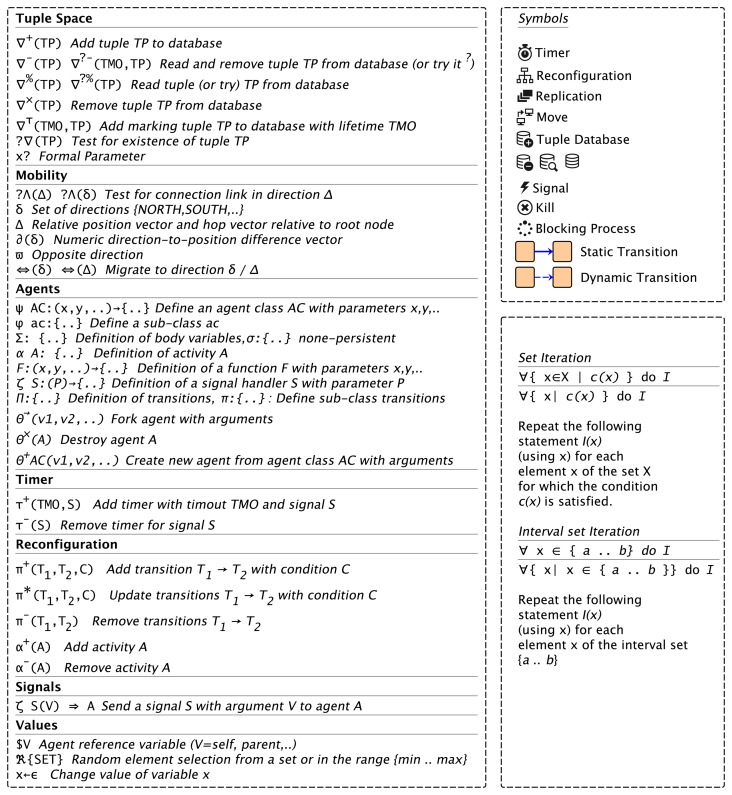
AAPL short notation and symbols related to the dynamic ATG and agent interaction model.

## A. Appendix

### A.2. Common AFL/AML FORTH Data Processing Command Words

**AFL**	**AFM**	**Description**

1	VAL(1)	Value literal word pushed on data stack
dup	DUP	Duplicate top element of data stack
rot	ROT	Rotate the three top elements of the data stack
swap	SWAP	Swap the two top elements of the data stack
>r	TOR	Move the top element of the data stack to the return stack
r>	FROMR	Move the top element of the return stack to the data stack
@	FETCH	Store the value of a variable (memory) on the data stack
!	STORE	Store top of the stack in a variable (memory)
+	ADD	Add the two top elements of the data stack and store result on the stack
−	SUB	Subtract the two top elements of the data stack and store result on the stack
*	MUL	Multiply the two top elements of the data stack and store result on the stack
>	GT	Compare the two top elements of the data stack and store result on the stack
<	LT	Compare the two top elements of the data stack and store result on the stack
=	EQ	Compare the two top elements of the data stack and store result on the stack
